# The Port-Folio; or, Medical and Surgical Miscellany

**Published:** 1819-01-01

**Authors:** 


### FOURTH DEPARTMENT.

THE

### Port-Folio;

OR,

### MEDICAL AND SURGICAL MISCELLANY.

### *Observations and Reflections on the reigning Epidemic or Typhous Fever.*

"Inter aulas ACADEMI querere verum."

*To the Editor of the Medico-Chirurgical Journal.*

SIR,

KNOWING how deeply you are interested in the cause of medical science, and with what attention you have yourself investigated the nature and treatment of fever, I shall make no apology for soliciting a few pages of your valuable Journal for the promulgation of the following observations and reflections.

Notwithstanding the light which has been shed on the subject of this paper by men of genius and experience in every age, from the days of Hippocrates till the present moment, it is evident, both from the writings and the discussions of the leading authors, teachers, and practitioners of the day, that a great difference of opinion, theoretical and practical, still exists among those few who, by their talents or conspicuous stations in society, are destined to guide the opinions and practices of the many, and particularly of the rising generation.

A most animated discussion on typhus fever has recently occupied a medical school of the first celebrity in this metropolis; in which discussion three of the brightest ornaments of the profession exerted all their talents and ingenuity, each to support his own theory and overthrow those of his competitors. This discussion, Sir, at which you were probably present, will make a lasting impression on the excessively crowded audience which witnessed it; while the numerous students, from various quarters of the world, as well as from every part of the united kingdom, will long relate the particulars of that memorable controversy.

Now, Sir, I think it will not be uninteresting to your provincial readers, and to those also of the metropolis who were absent from the meeting, to see a fair and impartial sketch of the discussion, drawn up by a man who can lay his hand on his heart and truly say,

"Tros, tyriusve mihi nullo discrimine agetur."

Neither do I think that the parties concerned can have any rational objection to an ungarbled statement being thus recorded by an unbiassed witness, who can have no inducement

"To aught extenuate, or aught set down in malice."

The discussion was opened by a gentleman, whose depth of research and ingenuity of argument are acknowledged by the profession at large, not only as promulgated in a *capital* work on fever, published a few years ago, but through the medium of his lectures as a public teacher in this metropolis. Dr. Clutterbuck began by observing that we had but two means of ascertaining the nature and seat of fever, or indeed of any other disease; namely, the symptoms during life, and the effects of the disease after death. He remarked, that the first of these two means was infinitely more important and less deceptious than the second; since derangements of *structure* were only the consequences or *effects* of derangements of action or function, and that, in many cases death might, and actually did, take place *before* functional produced organic lesion; and of course, in such instances no cognizable traces of disease could be discovered *post mortem.*

On the other hand Dr. C. maintained, that the organic lesions found after death, only proved the *great extent* to which functional or morbid action had gone during the life of the patient; while their absence only shewed that life was extinguished by functional disorder *anterior* to the production of structural alteration.

After these preliminary observations, Dr. C. recurred to the immediate subject of discussion, and went on to prove that all the *premonitory* phenomena of fever, as well as those which accompanied the disease through its whole course, were indicative of an inflammatory state of the brain. This last-mentioned organ, being the source of all sensorial power, manifested its derangements in a variety of ways, both immediately before and during fever. In the nerves of sense this was peculiarly evident. Thus he had seen men who, for several days before the unequivocal attack of fever, experienced such preternatural acuteness in the sense of hearing, that the noise of a carriage sounded like a discharge of artillery. In the eye, the visual nerve very commonly evinced a similar increase of sensibility, under the form of intolerantja lucis. The functions of the olfactory and gustatory nerves are all deranged at the commencement of fever, and even the feelings all over the surface of the body are in a morbid state. Lastly, the power of the voluntary muscles is diminished from the impaired state of the nerves supplying these muscles. Even the various functions of the thoracic and abdominal viscera are deranged from sympathy with the brain. The head ache itself, was a symptom so universal in fever that he need not dwell upon it.

Dr. C. here remarked, that in every case of fever which he had witnessed, the *head* felt hot, even when other parts of the body felt cold; and this he considered a proof of increased *vascular* action, as in other inflammations.

The impaired state of the brain, as the organ of the *mind*, is also, in Dr. C's opinion, equally manifest in the beginning and in the course of fever. He contended, also, that the remedies of fever were the remedies of inflammation; thus affording a strong corroborating proof of the identity of the two diseases, the *seat* of the latter having been already shewn to be the brain. Dr. C. then enumerated and commented on the different remedies which modern experience had proved to be most efficacious in fever; as bloodletting, purging, digitalis, antimonials, &c.; all of which, it is well known, are the most powerful means we possess of subduing local inflammation. In this part of the discourse Dr. C. took occasion to express his most decided dissent from the opinion of Dr. Bateman, as promulgated in a late work, that antimonials are next to useless in the disease under discussion. In this dissent he was joined by the various other orators assembled on the occasion. Dr. C. made many judicious and interesting remarks on the appearance of the blood drawn in fever; observing that this fluid, though it might not exhibit, in all cases, the *buffy* or *cupped* appearance, yet that, on minute investigation, there would be found what he would term a *sizy* state of it, still indicating local inflammation in the system. He here also took the opportunity of remarking that, however medical men might differ on theoretical or *other* points of practice in typhus fever, yet that so general a consent obtained throughout the more enlightened members of the profession respecting the propriety of venesection, under proper limitations and restrictions in this fever, as to remove all shadow of doubt on this once litigated question.

Dr. C. stated the great benefit which he has observed to arise from the administration of *digitalis* in fever, as a restrainer of the inordinate action of the heart and arteries, thus relieving the cerebral inflammation, on which he conceived the fever depended. He also strongly advocated the propriety of *emetics* at the commencement of fever, as a powerful means of equalizing the balance of the circulation, and relieving the brain of its undue proportion of blood.

From the remedies, Dr. C. descended to the *post mortem* appearances in fever. He contended, that although derangement of function, or, in other words, *morbid action*, often destroys life in fever, and thus leaves no trace of altered structure in the brain; yet that, *most frequently* we *do* find unequivocal proofs of preceding inflammation, as evinced by effusion of serum or coagulable lymph between the membranes, or in the ventricles of the brain—by preternatural fullness of the vessels—by increased vascularity of the encephalon itself—by unusual firmness of this organ, and by evident thickening and inflammation of the coverings of the brain.

Dr. C. concluded by observing, that these were not mere matters of speculation, but that *theory*, which ever did and ever will obtain in medicine, exercised an imperious sway over the practice of our art. He exemplified this observation by the influence which theory had over practice in the exhibition of mercury for the cure of venereal diseases. When the doctrine of a morbid *matter* in the system, as the cause of the disease, prevailed, and consequently that its *expulsion* was the only cure, then profuse and long-continued ptyalism was excited by mercury. But when a more rational theory of *morbid action* was entertained, then a great revolution took place in the administration of the remedy. He therefore insisted that our practice would never be steady, or properly efficient till it was grounded on a rational and true theory. Till that time the *methodus medendi* must proceed on the plan of *relieving* or *curing* symptoms, instead of *meeting* and *preventing* them, which is infinitely preferable.

Dr. C. inferred, that if he was successful in proving fever to be an inflammatory condition of the encephalon, the division of typhus into "*simple*, *inflammatory*, *and congestive*," adopted by a late author, must fall to the ground.

The author of the division above-mentioned, Dr. Armstrong, rose to defend the classification he had adopted in a work which stands unrivalled on typhus fever. He stated that he would wave the subject of *post mortem appearances*, which Dr. C. had so ingeniously disentangled himself of, and take him on his own ground of symptomatology, as the surest index of what was going forwards within. Dr. A. now drew the most animated picture of congestive typhus that ever flowed from lip or pen. The whole assembly were as mute and motionless as though they had been statues. He appealed to Dr. C——, he appealed to every unbiassed auditor whether a disease setting in with overpowering lassitude—feebleness of the limbs; giddiness of the head; dingy pallidness of the face; anxious breathing; damp, relaxed, or dry withered skin; coldness of the surface; quick, weak and trembling pulse; glary, vacant eye; and ending fatally in a few hours, without the slightest symptom of reaction or excitement, could possibly be set down, as a case of *inflammation of the brain?*

Dissection, in such cases, would show only engorgement of the *venous* system, but no vascularity of the arteries; no effusion of serum or coagulable lymph; no hardness of the brain; no redness of its membranes. These *post mortem* proofs of the non-existence of inflammation, however, he would not dwell on, since Dr. C. evaded them; but he would ask Dr. C. were the *symptoms* above described, those of inflammation of the brain? Were they not symptoms of a state the very *reverse* of inflammation.

If, (said Dr. Armstrong) a man fell down in the street, while a cart wheel passed over his arm, and crushed it to pieces; if this man, as he most probably would, on being taken up, exhibited a pale countenance; fluttering pulse; interrupted respiration, and confusion of thought; would any surgeon set these symptoms down as the incipient phenomena of the inflammation which would shortly succeed in the lacerated member? Yet surely Dr. C. in attributing the symptoms of the first stage of oppression and of the second of reaction to *inflammation* of the brain, was acting under this erroneous impression.

Dr. C. had stated that a judicious practice must flow from a sound theory. He, Dr. A. admitted this; and he would try Dr. C's theory by the criterion in question. If that stage of oppression, which ushered in many dangerous forms of fever, were dependent on *inflammation* of the brain; surely, upon this principle, we ought to treat it by the usual means resorted to in cerebritis—viz. blood-letting, purgatives, &c. But what would be the result of this practice? inevitable death to the patient! Dr. A. therefore insisted on the propriety and the practical utility of dividing fevers into stages and classes, affirming that the phenomena, the treatment, and the dissections, all conspired to show that the pathology of what he described as congestive fever, consisted in congestion of blood in the *venous* system; a state the very *reverse* of inflammation, and which was, in fact, cured or removed by the subsequent *excitement*, which might or might not end in *inflammation.* He acknowledged, however, that the *brain and spinal cord* were the organs on which the onus of the disease chiefly lay during life, and which most frequently displayed the effects of fever after death.

Of the simple typhus, Dr. A. considered the proximate cause to be excitement of the heart and arteries, without any local determination, congestion or inflammation, at the beginning; and in this light, the Doctor seemed to view what he denominates *inflammatory typhus, in his primary movements,* the local inflammations or congestions being secondary, and resulting from *local* predisposition in the organ or part itself, which becomes the seat of inflammatory action or engorgement during the general febrile commotion in the system.

When Dr. A. had ended his reply, a gentleman of distinguished talents, and many years an eminent teacher in the theatre where the discussion was held, arose. Dr. Curry commenced by declaring that though now both old and grey in the exercise of his profession, he would yet " put his satchel on his back," and come to take a lesson from his junior brethren. He appealed, however, *in limine*, to every one present, who may have heard his lectures in that theatre, whether he had not been in the habit of teaching and inculcating the doctrines and practices (with very trifling shade of difference) of the last speaker, [Dr. Armstrong] for nineteen years past, as might be seen and proved also by his Syllabus of Lectures. Nevertheless, he ventured to assert, in the face of all the evidence adduced, that the present epidemic fever was *not* typhus ;* nor to the best of his knowledge, had he seen a single case of typhus during the last twenty years. Dr. Curry stated that, soon after his return from the East Indies, about (I think) the year 1793, he had an opportunity, in conjunction with his predecessor, then Physician to Guy's Hospital, of seeing fever in all its shapes, throughout this great metropolis. But the typhus of that period, and the so-called typhus of the present day, had no similitude or affinity, save the *name*, which, in the latter case, was erroneously applied. He positively asserted that the typhus fever, so admirably described by Huxham, in which the patient goes crawling about for five or six days, " seemingly not very sick, and yet far from being well," with a heavy, pale, and sunk countenance—where, about the seventh or eighth day, the head-ache and giddiness, the load on the præcordia, anxiety, and faintness, grow much more urgent—where actual deliquium commonly occurs on attempting to sit up, with continual muttering and faultering in the speech—where the stools, urine, and tears run off involuntarily, ultimately ending in death, on the 14th, 18th, 20th, or even 30th day—such a fever, Dr. Curry averred, was now no where to be seen.

Here Dr. Curry took occasion to pass an eloquent, and not unmerited eulogium on our own immortal Sydenham, whom he looked upon as little inferior to Hippocrates himself. The longer Dr. Curry lived, and the more he saw, so much the more strongly was he convinced of the truth of Sydenham's opinion, that diseases obeyed a periodical influence, partly from the circumambient air, and partly from terrestrial exhalations, or from both combined. He supported this opinion by many ingenious reasons, and important facts, drawn from the histories of epidemics since the year 1793.

---

* See a striking coincidence of opinion between this eminent physician and Dr. Porter of Bristol, as contained in a very interesting Pamphlet on the reigning Epidemic Fever, just published by the last-mentioned Gentleman.—Ed.

Among other data on which he has grounded his assumptions, the following one was stated. In the autumn and winter of 1809, when the Walcheren fever produced such havock among our troops in the Islands of Zealand, Dr. Curry affirmed, from his own personal knowledge and observation, that there was not a street or lane in this metropolis wherein remittent and even intermittent fever might not be seen among the inhabitants. Hence he concluded, that the same aerio-terrestrial influence, which showed itself so fatally in the debouches of the Scheldt, where vast bodies of men were congregated under circumstances exceeding favourable to its full operation, was also acting with greater or less power, over England, and probably over the whole surface of the globe. It was in this way, he said, and upon this principle only, that the spread of those desolating epidemics, which in some years, ravaged the western tropics, and the countries of North America and Europe partaking of a tropical nature, could be rationally accounted for. This view of the subject led him to suspect that many young men of the present day, who prided themselves on having discovered the true nature and the most effectual treatment of fever, and who supposed that their forefathers and predecessors were all in the dark, in these respects, had, in reality, but little cause for self-gratulation, since the diseases which their despised forefathers treated in this supposed unskilful manner, were very different from those of the present day; and hence that it was very probable—nay, that it was almost certain, that future generations would eye the doctrines and practices of our times with as much astonishment, and as exultingly contrast them with their own, as we do now with respect to times past. This consideration, he maintained, ought to repress the pride of the present generation.

Into the doctrine and practice of Dr. Armstrong he would not enter, since he had taught them in the broad outline (with the exception to the name of *typhus*) for so many years; but he would just say, in a few words, that one or two copious bleedings at the onset of re-action, with purgatives, local detractions of blood, a grain of calomel every four hours, and three or four grains every night, in general conducted these erroneously denominated "*typhous fevers*," to a safe termination in the course of five, six, or seven days, another proof how greatly they differed from the real typhous fevers of Huxham, Pringle, &c.

As to Dr. Clutterbuck's theory of fever having its *fons et origo* in inflammation of the brain, it was utterly untenable by any man who did not view the phenomena of the disease through a distorted medium. The causes which produced fever must act on the external or internal surfaces of the body, and affect the brain as a *passive* organ, in the same way as they affect other organs, through the medium of the nerves and blood-vessels. It was therefore no more the *exclusive* or primary seat of fever than the stomach, intestines, liver, lungs, or any other structure or tissue in the body. This was proved by the phenomena during life, and the appearances after death.

Dr. Armstrong again arose and made a short rejoinder to the observations of the last speaker. He did not conceive that he had occasion to touch on any part of the learned and ingenious physician's speech, except that wherein it was asserted, that we had no such thing as typhus among us in the present day. He would aver, however, from repeated personal observation in the fever hospital of this metropolis, that scarcely a day passed, in which cases, exemplifying every ioto of the descriptions drawn by Huxham, and by Dr. Curry himself, did not present themselves at that Institution. He could not, therefore, but consider the scepticism of Dr. C. on this point, as unsupported by the observations and experience of the great majority of medical practitioners. He was happy, however, that he lived in a period when medical men could differ from each other *in opinion,* without the least diminution of reciprocal *esteem.*

Dr. Clutterbuck rose, but without any intention of vindicating, by any farther arguments, the truth of the theory which he had advanced regarding the primary seat of fever in the brain. He left the subject to time, and to the adjudication of the public voice at large. But he could not help expressing his astonishment at the assertion of Dr. Curry, that *typhus* fever had not existed in this metropolis during the last twenty years.* He fearlessly asserted, that he had repeatedly witnessed, during the present epidemic fever, all those traits and phenomena which have been so faithfully and accurately described by Sydenham and Huxham. He therefore begged leave to dissent, in toto, on this point, from the learned physician who had lately addressed the society.

Here several members of the society, many of them men of solid judgment, extensive experience, and accurate observation, gave evidence in favour of the reigning epidemic fever of the present day being a typhus fever, and contagious. Among others, a gentleman distinguished for sound practical knowledge, scientific acquirements, and considerable powers of elocution, brought forward some very HARDY proofs in favour of the typhous character of the present epidemic fever, and also in favour of the present depletory plan of treatment, as contrasted with that which was pursued twenty years ago.

He stated that, so long ago as the year 1798, the very period in question, he was in the habit of visiting the typhus patients in Guy's Hospital, under the care of the late Dr. Saunders, and could vouch for the identity of those and the present fevers. The results of treatment, however, were very different; for at that period, the success was little and the mortality great; whereas it is now just the reverse. The practice was then stimulating—it is now deple-

---

* The Editor of this Journal, who heard the discussion, begs to bring to the recollection of Dr. Clutterbuck, that Dr. Curry admitted the *occasional* existence of typhus among the dregs of society in the purlieus of St. Giles's and similar places.

tory. Of course, however similar might be the fevers in their *early* stages, the symptoms of the *ulterior* stages were necessarily modified by the difference of treatment. But still they were the same fevers, derived from the same causes, propagated by the same laws, and equally fatal or mild, according to the treatment pursued.

In the course of this most interesting discussion, on a former evening, a vast body of evidence came out, furnished by this and several other gentlemen of talents and experience, tending to prove that the fever in question very often originated, de novo, from crowding, uncleanliness, want of ventilation, and mental and corporeal misery. That whether it originated thus, or from direct and unequivocal contagion, it propagated itself afterwards infectiously, unless the various and well known means for checking contagion were rigidly put in force. It is true that several counter-statements were made in opposition to this; but they were, with only one or two exceptions, from young men who had little practical knowledge of the disease, and who grounded all their arguments on the ingenious, perhaps in some places, erroneous doctrines of Dr. Bancroft.

The whole mass of evidence was in favour of depletion, vascular and intestinal, in the *early* stages of the fever, even in this metropolis, where so general an opinion had so long prevailed respecting the inability of citizens and sedentary mechanics to bear these measures. They all seemed to argue also, that where early evacuations were employed, there was seldom occasion to have recourse to stimulants or cordials in the close of or convalescence from the disease.

Upon the whole, Sir, I believe you will allow that the society exhibited, during this memorable discussion on fever, a body of talent, an extent of information, a display of science, and a degree of zeal amounting almost to enthusiasm, which augur well for the advancement of the medical art, and do infinite honour to its present cultivators, both old and young.

And now, Sir, after stating the arguments of the different orators, may I be permitted to make a few observations and reflections of my own.

The first question which naturally arises out of the foregoing discussion is that which bears on the *local* or general nature of idiopathic fever.

If it be granted that fever depends on a *local* inflammation, as of the brain, then the division of the disease into *stages* must at once be given up. Do we, or can we, divide inflammation, in any other case, into stages with propriety? Is not inflammation the *same* till it *ends* in resolution, suppuration, gangrene, &c.? But are the phenomena of fever the *same* till some termination of the disease?—No indeed. We have, in general, a first stage, whose phenomena are just the reverse of those in the second. Instead of *heat* on the surface, we have *cold;* instead of a full hard pulse, we have a small, weak, and quick one; instead of the arterial capillaries being injected beyond their usual size, they are deserted of blood, and the whole external surface of the body is collapsed, while the glandular or secreting organs are, as it were, dried up. According to the *cerebral* doctrine, this is inflammation of the brain just as much so as in the subsequent stage of re-action. If so then, why not check the inflammation in this stage, by bleeding, purging, starving, and all those means which we know are useful in *every stage* of inflammation till its *termination*? In what possible way can the abettors of *local* inflammation, as the *primary* essence of fever, get over this question?—In no other way, that I can see, but by admitting that the inflammation in fever is *different* from other inflammations—and then we are just where we started!

Whether we look at the phenomena during life then, or the sequelæ after death, we must acknowledge that even where local inflammation unequivocally obtains in fever, a state or stage preceded that inflammation, presenting quite other symptoms, and requiring quite other treatment. The doctrine then appears to be wrong in principle, and dangerous in practice. It breaks up the catenation of cause and effect, or ratio symptomatum in fever; it annihilates the important divisions of stages, and points to *one* essential mode of treatment, which every practical observer knows is inapplicable, nay, injurious in the different stages which nature herself has clearly chalked out.

In respect to the doctrine of periodical changes in fever, resulting from ærio-terrestrial influence, I think it has been rejected without sufficient examination. Having lived nearly half a century in this world, I am decidedly of opinion that fevers, and many other diseases, have undergone considerable revolutions in their *nature* as well as in their treatment; but whether this change be *solely* owing to ærio-terrestrial influence I am not quite so certain. Something, I think, might be put down to the account of progressive civilization and moral refinement. But whether this be granted or not, the fact itself cannot easily be denied. Can we look at the present nature and treatment of syphilis, as contrasted with both twenty years ago, and not be convinced that the hand of time works great changes on all terrestrial phenomena. And here I may remark on a position which was pretty confidentially advanced from all sides of the house in the late meetings; namely, that the lessened ratio of mortality in the present time, as contrasted with the past, is solely owing to the improvement in the treatment of fever. I do not admit it. I believe that the lessened mortality in fever is as much, if not more owing to the improved condition of those among whom it is found, than to any difference of treatment. Indeed, I am strongly disposed to think that, provided no injurious or *stimulating* measures were employed in fever, the proportion of mortality would not be much increased or altered, whatever other means were used, and however opposite in their kind.

As to the doctrines and practice adopted by Dr. Armstrong, they appear to come very near the true state of things; yet I would venture, with due deference, to dissent a little from that distinguished and popular physician, "without any diminution of esteem," to use his own words. I would say that Dr. A. has looked too much to the vascular and too little to the nervous system in his doctrines; and that although he combats the theory of local or cerebral inflammation as the cause of fever, he yet gives too much countenance to it in his writings. Speaking of the contrast between congestive and inflammatory typhus he admits that "possibly something similar [to inflammation] may now and then take place in what has been called the congestive typhus, an *actual inflammation* being masked under external appearances of a *deficiency* of general heat and of arterial tone," &c. 72. Dr. Clutterbuck might have taken advantage of this passage, and turned it to the support of his own doctrine.

Permit me, Sir, in conclusion, to throw out a few theoretical and practical hints of my own.

I am strongly disposed to think then, that all the phenomena, premonitory, and coexistent of *incipient* fever, indicate a diminished energy of the *nervous* system; and that the consequent diminution of energy in the *heart*, leads to a turgid state of the venous system, because that organ cannot keep up the balance between the veins and arteries. Now the nervous system having received the first shock from the cause or causes of the fever, whatever they may be, is thereby debilitated; and I believe it will be found that towards all debilitated organs or parts, there is naturally an afflux of blood, whenever the balance of the circulation is disturbed. In this case then, the venous vessels of the brain and spinal marrow become turgid with blood, and the pressure on the sources of sensation and volition disturbs the equal distribution of sensorial or nervous energy, whence the state of the secretions and temperature, so dependent on the nerves, becomes greatly deranged, or, as it were, suppressed. All these phenomena result from the operation of the morbid cause on the nervous and vascular systems; and most of the subsequent phenomena included in the term *reaction*, are sanative efforts of the constitution to restore the lost balance of sensorial distribution and sanguineous circulation. And I do maintain that our best rules of treatment hinge on this supposition of morbid impression and salutary reaction. During the *first* we are often obliged to give cordials, use the warm bath, &c. During the *second*, our sole efforts are directed towards the regulation of nature's movements; to restrain them when inordinate, and excite them when deficient.

And, Sir, after all the precise and formal codes of rules and restrictions which have been framed for us by authors and teachers on the subject of fever, I have never found any so generally applicable and so truly practical as the ancient one of Hippocrates—*Contraria contrariis medentur*, or as it has been termed and ridiculed —"Obviating occasional symptoms." What more do the best practitioners of the present day? If we see a man who has been exposed to the influence of contagion or any other efficient cause of fever, with pallid face, dejected air, cool skin, constipated bowels, and, in short, all the phenomena of the primary morbid impression, what are we to do? why little else, if symptoms are moderate, than clear the bowels, avoid food, and wait for the reaction of the constitution. But if the morbid impression have been so powerful, the action of the heart so weak, and the distribution of sensorial energy so inefficient, as to give reason to fear that the vessels of the brain, the spine, and the glandular organs, may become gorged with stagnant venous blood to an irremediable extent, what do we? We solicit the blood to the surface by the warm bath; we exhibit medicines that are known to have a tendency to rouse the action of the heart, equalize the circulation, and diffuse the vital power of the nervous system—as warm cordial drinks, opium, and calomel, or even emetics. If these fail, what are we to do? We know that the vital organs are overpowered with semi-stagnant blood, and we want reaction, we even prefer *inflammation* to the present state. In such a desperate situation, the *anceps remedium* is better than none. Blood must be taken, even at the risk of life, to free the oppressed viscera; but we here neglect too much the local abstractions of this fluid. We should cup in the back of the neck and along the spine; and then apply blisters to the same parts. If these fail, blood must be taken from the system at large; not upon the principle of checking inflammation, but with the anxious hope of bringing on that reaction of the system, in which stage or state only, inflammation is apt to occur.

When Nature, alone or assisted, has brought on reaction, our business is to restrain it within due bounds. If general arterial excitement runs high, we are to lessen the action of the heart by general blood-letting, proportioned to circumstances, and we are to watch the weakest organ, and whenever a demonstration is made towards it, we are to curb it by local or general evacuations. We see, in most cases, secretions and excretions deficient and morbid; and the rule above mentioned directs us to the increase and amelioration of these, by purgatives and mercurials. If, towards the close of the contest, the powers of the constitution flag, from previous excessive action, we must endeavour to sustain them by cordials, especially wine, proportioned to the necessity of the case. In short, Sir, I believe that every observant practitioner, who carefully watches the morbid impression, and sanative reaction, will be able, with little difficulty, to find out the *indications* to be pursued in fever; and that if he promptly check each inordinate movement in the system, and be ready to assist Nature when deficient in power, he will practise more successfully, in the end, than if he act on the principle of taking the work entirely out of Nature's hands, by attempting to arrest, *in toto*, the disease by violent and artificial means.

I have the honour, Sir, to subscribe myself,
Your humble Servant,

ATTICUS.

London, December 25th, 1818.

### II.

***Report of Diseases in the* UNIVERSAL DISPENSARY FOR CHILDREN, *St. Andrew's Hill, Doctors' Commons, from September* 1, *to December* 1, 1818.**

In the early part of September, a great many cases of fever, chiefly resembling Synochus, were noticed among the children that were brought to the Dispensary, from two to ten years of age. It attacked with the usual symptoms of nausea, vomiting, shivering, flushed countenance, heaviness of the eye, redness of the conjunctiva; shortness of breathing; tenderness; pain and oppression at the præcordia; pain in the head, which was frequently intense; excessive thirst and prostration of strength. In some of the patients, the skin had a yellow tint from the commencement of the attack; and in others, it became so in the progress of the fever, though it was by no means an invariable appearance. In the majority of instances, the alvine discharge was considerably diminished, the tongue uniformly covered with a thick white or yellow sordes; the pulse rapid, strong, and bordering upon hardness; the urine small in quantity and high coloured. The children thus attacked were in a state of continued restlessness and jactitation, and in a few hours they became delirious, screaming out very often from the severity of pain in the head. This fever had no perceptible remission, even after five or six days' duration, at which period of the disease the patients generally became comatose, respired with a slight stertor, and when roused, seldom so far recovered their senses as to recognize the persons around them; or, if they did, instantly screamed out, then moaned, tossed themselves about involuntarily, and appeared to experience distressing and painful anxiety. In the protracted cases of this fever, all the well-marked symptoms of Hydrocephalus supervened; and dissection proved that congestion had existed to a great extent in the brain; that effusion in the ventricles, and between the dura and pia mater had been the immediate causes of a fatal issue.

The viscera of the abdomen bore however, in these cases, evident marks of active inflammation, particularly the stomach and intestines; and the liver was unusually large, and of a firmer texture than natural.

Frequent opportunities presented themselves, at this season, to notice, among children that were brought to the Dispensary, considerable derangements in the hepatic function, under which their health visibly declined, though no disease of a specific character had, as far as could be detected, formed in the system. Progressively, they became languid, feeble, and irritable; the skin grew sallow; the abdomen large; the food was rejected; now and then diarrhœa came on. The stools seldom contained any admixture of bile, and either resembled jalap mixed with water, or the paste of oatmeal, and always had a peculiar fœtor. These cases were not easily relieved, and often degenerated into true Synochus. Even where an early opportunity had been given to treat these hepatic derangements, the biliary secretion would continue irregular and vitiated for two or three months, one day appearing in excess and highly acrid, another day being suspended or passing off in green viscid drops, separate from the fæces. Neglected instances of this affection degenerated into colliquative diarrhœas and dysentery, and the patients died hectic.

Enteritis, Cholera Morbus, Dysentery, and Diarrhœa, were the more uniform diseases in October: but, in November, Synochus, in all its various modifications, especially that form of it in conjunction with Phrenitis, was by far the most common acute disease among children, from two to ten years of age.—The Hooping-cough now also began to prevail with considerable severity; Catarrhal Affections and Pneumonia, the Scarlet Fever, Roseola Autumnalis, Varicella, and Urticaria. Many cases of Acute Rheumatism were likewise noticed in infants under two years of age. One child, nine months old, had this disease, in its most acute form, in the shoulders, wrists, and knees. Phrenitis appeared, often as a modification of fever, sometimes in connection with dentition, and was attended with convulsions and strabismus. During the three months embraced in this Report, Hydrocephalus Acutus and Chronicus, were, as usual, frequent diseases. Neither was the season free from erysipelatous affections, though they were, upon the whole, mild, with the exception of one case of a boy, eleven years of age, who had an attack of Erysipelas in the right leg and thigh, which extended from the pubis to the toe, accompanied with considerable extravasation in the cellular membrane, independent of large and diffused vesications on the surface. He had high pyrexia, a feeble and rapid pulse, a black tongue, and was comatose throughout every stage of the disease. After continuing four weeks, it produced a permanent contraction of the flexor tendons of the muscles of the leg and thighs; but the boy recovered his health.

A case of a child, with a periodical Fever, and an appearance of bullæ of the size of a small almond on the body and lower extremities, connected with a dyspeptic state of the stomach and bowels, has baffled every treatment for months. Successive bullæ appeared upon each attack of fever. The child grew hectic; but is at present in the country, under more promising circumstances.

Since the latter end of October, all the acute diseases of children have been exceedingly severe, and at the time of writing our Report, the character of severity is daily heightening: Fevers, Visceral Inflammation, Measles, Hooping Cough, all prevailing under an aggravated form. Cynanche Trachealis has occurred frequently; Cynanche Parotidœa and Scarlatina are reigning diseases at this moment.

John B. Davis,

William Shearman, } Physicians.

December 12, 1818.

*The Table of Diseases, (521 Cases) was omitted for want of room.* Edit.

### III.

### Dr. Grattan's *Report of the Fever Hospital, in Cork Street, Dublin.*

[ Transactions of the King's and Queen's College. ]

In the first Number of this Journal, first Edition, page 70, by some unaccountable oversight, we attributed the sentiments of Dr. Grattan relative to Fever, to Dr. John O'Brien. We here beg leave to correct this mistake, and to give a part of the passage entire, in the words of Dr. Grattan himself.

" Synocha, in its derivative and proper signification, implies simply, continued fever; and if employed in this sense only, it will denote fevers of the first class, in which no disturbance of the sensorium, or appearance of local inflammation is found to exist. When to the symptoms of simple fever, there are superadded those which indicate inflammation, the termination *itis* will mark the existence of such inflammation. Thus synochitis comprehends fevers of the second class, which are distinguished by local pain and inflammation of one or more organs, the sensorial functions remaining undisturbed. Of this division, the genera are as various as the organs affected, and may be termed pleuritic, gastric, enteritic, hepatic, &c.

" Typhus originally signified fever with stupor; but it has so far departed from its original meaning, that it is now generally used for every form of contagious fever, which assumes a malignant character. In fact, it is a term at present so indefinite, and so universally misapplied, that its abuse has created the greatest confusion, and contributed materially to retard the improvement of medical practice in fever. Hence, instead either of restricting it to fevers attended with stupor, or of extending it to all which exhibit a malignant appearance, I think that it should rather be employed to denote those fevers which are accompanied with an evident disturbance of the sensorium, without any symptom to indicate inflammation. In this sense, it will include the fevers of the third class, and still be used as heretofore, in contradistinction to synocha.

" By changing Typhus into Typhitis, we obtain a term adapted to explain the nature of the fourth and last class, to which belong all Fevers that present Typhous symptoms, complicated with inflammation of one or more organs. The genera of this class are similar to those of the second, and are formed on the same principle. In both, the local inflammation constitutes the generic difference, and the generic names of each are consequently the same.

" By retaining Synocha and Typhus in a strict and determinate sense, and employing Synochitis and Typhitis, to denote the existence of local inflammation, when superadded to the primitive form of each, it appears to me that the practitioner may be assisted in determining with greater certainty and facility, the treatment most likely to prove beneficial. In Synocha, purgatives are mostly to be depended on. In Synochitis, in addition to purgatives, blood letting and blisters must be employed. In Typhus, purgatives, blisters, cordials, and sometimes topical blood-letting, are necessary; and, in Typhitis, purgatives, blood-letting, blisters, and occasionally cordials, are indicated."

*Transactions, page* 484.

---

### IV.

### *High Operation for the Stone.*

There was lately performed, on a boy of about 14 years of age, at St. George's Hospital, an operation for the Stone, wherein the calculus was removed by an incision above the pubes. The steps of this process were, we understood, as follows:—The patient being secured in the usual manner, a sound was introduced into the bladder, and an incision was made into the membranous part of the urethra through the perineum. A director was then introduced into the bladder, and the staff withdrawn. A kind of bistoire cachée, in the shape of a catheter, was next introduced along this director, and the latter removed. The bistoire being pushed up so as to press upon the upper part of the bladder, an incision was made in the linea alba, in the place where we puncture the bladder; but this incision was not carried right into the bladder, the cutting part of the bistoire cachée was pushed forward and made to come out at the wound in the linea alba. This wound was next enlarged downwards towards the pubes, by a bistoury, the point of which, we understand, was fixed into the groove of the bistoire cachée. The stone, it seems, was encysted, and Sir Everard Home was obliged to introduce his finger to free the calculus, which was removed by the forceps, though with some difficulty. The bistoire cachée being removed, a hollow gum catheter was introduced through the lower wound into the bladder, so that the water might drain off constantly, and prevent extravasation from the upper wound. The boy has had a remarkably good recovery, without any sinister accident.

---

### V.

### M. Broussais's *Conversion from the stimulating to the depletory Practice in Typhus and other Fevers.*

During the treatment of more than 400 cases of acute fevers, many of them what are denominated Typhus, I have not had, in the last six months, to attribute a single death to any thing but clandestine imprudence, or non-obedience on the part of the patient. The effects of this non observance of my directions were so striking, as to convince my pupils of the propriety of the plan which I pursued, and of the danger of stimulation. The general practice of this country I departed from, in consequence of being wearied with beholding every symptom exasperated by tonics; while traces of inflammation were seen in the viscera of every patient that died. I ventured, at first, on depletion with great caution; but, emboldened by success, I clearly discerned, at last, that the external marks of *debility* were but the faithful expression of morbid irritation in some *internal* viscus. From multiplied and extensive experience, I have come to the following conclusions: 1st. That Typhus is not so common a disease as many people would persuade us to. 2nd. That the majority of fevers are accompanied, or rather caused by a *sub-inflammatory* state of the mucous tissues of the abdominal viscera, which is aggravated by emetics and tonics, and easily removed by antiphlogistic measures. 3d. That symptoms of debility and putrescency only show themselves where Nature is left to exhaust herself in useless or superfluous efforts; or where the irritation, on which depends the febrile state, is augmented by improper remedies. 4th. That during the period of excitement, in real, contagious typhus, it ought to be treated as an inflammation of the digestive apparatus, by rigid abstinence, diluents, and even general and local blood-letting, whenever the visceral irritation threatens disorganization of structure.

Cerebral and pectoral irritations are common in typhus. The *first* is generally consecutive of that in the digestive organs; but not seldom primitive, especially in spring and summer. The pectoral irritation is more frequently developed in winter, and occasions that vast mortality observable in the typhous epidemics of cold countries.

These typhous phlegmasiæ offer no fair contra-indication to antiphlogistic measures. Thus cerebral irritation warns us to apply leeches to the temples, or course of the jugular veins; to keep the head encircled by cold applications; to immerse the lower extremities in warm water; to open the bowels; to produce revulsion from the encephalon by sinapisms, blisters, &c. It is not, in short, by applying diffusible stimuli, as the Brunonians do, to all the organs without distinction, that any good is done by tonics or other irritants in typhus. It is by stimulating a torpid organ or part that we lessen irritation or excessive excitement in another organ or part of the system.

---

We have been much pleased to see a new and very interesting little periodical publication, which is distributed to the profession *gratis*, by Messrs. Burgess and Hill, 55, Great Windmill-street, entitled, the Medical Intelligencer, or Monthly Index to the periodical Publications of the present day. It is got up with great care and labour, and presents an Index of Medical matters, the extent of which will create astonishment in the mind, and convince the most sceptical that Medical science is at this moment cultivated with an ardour far surpassing that of any other period in the history of Medical literature.

---

Mr. Curtis, the Surgeon of the Royal Dispensary for Diseases of the Ear, has lately found gentle emetics highly serviceable in cases of incipient deafness in young Children.

Mr. Clarke's and Mr. Blagden's next Course of Lectures on Midwifery and the Diseases of Women and Children, will commence on Monday, January the 25th.—For Particulars apply to Mr. Clarke, 10, Saville Row; Mr. Blagden, Duke Street, St. James's; or to Mr. Stone, 2, Upper John Street, Golden Square.

Mr. Curtis will commence his next Course of Lectures on the Anatomy, Physiology, and Pathology of the Ear, on Thursday, January the 7th, at seven o'clock in the evening, at the Royal Dispensary, Carlisle Street.

Westminster Eye Infirmary.—*Lectures on the Practice of Physic.*—Dr. C. F. Forbes, Deputy Inspector of Military Hospitals; Physician to His Royal Highness the Duke of Kent; Senior Physician to the Surrey Dispensary; Physician to the Royal Westminster Infirmary for Diseases of the Eye, &c. will commence a Course of Lectures on the Practice of Physic, on Wednesday, January the 7th, at nine o'clock in the morning, at the Royal Westminster Eye Infirmary, Mary-le-bone Street, Golden Square.—Terms of Attendance. Single Course, *Three Guineas.* Perpetual, *Five Guineas.* Medical Officers of the Navy, the Army, and the Ordnance, will be admitted to attend these Lectures, on presenting a recommendation from the Heads of their respective Departments, to *Dr. Forbes*, at his house, No. 25, Argyll Street, at nine o'clock in the morning.

*Mr. Guthrie on Surgery.*— Mr. Guthrie, Deputy Inspector of Military Hospitals, will commence his Spring Course of Lectures on Surgery, on Monday, January 18th, at five minutes past eight in the evening, in the Waiting Room of the Royal Westminster Infirmary for Diseases of the Eye, Mary-le-bone Street, Piccadilly. To be continued on Mondays, Wednesdays, and Fridays.—Two Courses will be delivered during the Season. In each Course the Principles of Surgery will be explained, and the Practice resulting from them, with reference both to Domestic and Military Surgery, fully pointed out. The Diseases of the Eye, although forming an integral Part of the Lectures on Surgery, will, for the convenience of illustration, be delivered every Thursday evening until completed. The Operations referred to in the Lectures will be shewn in each Course.—Terms of Attendance. Perpetual, *Five Guineas.* Single, *Three Guineas.* Medical Officers of the Navy, Army, and the Ordnance, will be admitted gratis, on obtaining a recommendation from the Heads of their respective Departments, which must be presented to *Mr. Guthrie* between the hours of two and half-past four, at his house, No. 2, Berkeley Street, Berkeley Square.

Dr. P. M. Latham and Dr. Southey will begin their Course of Lectures on the Practice of Physic, on Monday the 25th of January next, at the Middlesex Hospital, at nine o'clock in the morning.

### NEW WORKS.

Just published, Elements of Medical Logick; illustrated by Practical Proofs and Examples. By Sir Gilbert Blane, M.D. Physician to the King, &c. &c. &c. 8vo.

Practical Illustrations of the Progress of Medical Improvement for the last Thirty Years; or, Histories of Cases of Acute Diseases, as Fevers, Dysentery, Hepatitis, and the Plague, treated according to the Principles of the doctrine of Excitation, by himself and other Practitioners, chiefly in the East and West Indies, in the Levant, and at Sea. By Charles Maclean, M. D. &c. 8vo.

Illustrations of the Power of Emetic Tartar in the Cure of Fever, Inflammation, and Asthma; and in preventing Phthisis and Apoplexy. By William Balfour, M. D. Author of "A Treatise on Rheumatism, &c."

In the Press, A Treatise on Midwifery, enforcing new principles, which tend materially to lessen the sufferings of the Patient, and shorten the duration of Labour. By John Power, Accoucheur, Member of the Royal Medical Society of Edinburgh.

An Account of the Epidemic and Sporadic Disorders which prevailed this year, 1818, at Rochester, and near it. By Walter Vaughan, M. D. Licentiate of the Royal College of Physicians of London. 8vo.

Illustrations of the Power of Compression and Percussion in the cure of Rheumatism, Gout, and Debility of the Extremities; and in promoting general Health and Longevity. By William Balfour, M. D. Author of "A Treatise on the Sedative and Febrifuge Powers of Emetic Tartar, &c. &c."

In a few weeks will be published, No. I. of a new System of Medical Botany, handsomely printed in royal octavo, with plates coloured according to Nature, and Botanical Descriptions of each Plant, giving the place of its Growth, Medical Properties, Doses, &c. The Work is intended to comprise all the Plants now used by Medical Practitioners in the Materia Medica of the London, Edinburgh, and Dublin Pharmacopœias.

Transactions of the Association of the King's and Queen's Colleges, Dublin. Vol. II. are just published.

Remarks on the Prevailing Epidemic, or Typhus Fever of the United Kingdom, by William Ogilvie Porter, M. D. of Bristol, sewed.

